# Extracellular Vesicles as Potential Therapeutics for Inflammatory Diseases

**DOI:** 10.3390/ijms22115487

**Published:** 2021-05-22

**Authors:** Hee Sook Hwang, Hyosuk Kim, Geonhee Han, Jong Won Lee, Kwangmeyung Kim, Ick Chan Kwon, Yoosoo Yang, Sun Hwa Kim

**Affiliations:** 1 Center for Theragnosis, Biomedical Research Institute, Korea Institute of Science and Technology (KIST), Seoul 02792, Korea; hiya1092@gmail.com (H.S.H.); hyoseog7@kist.re.kr (H.K.); geonhee@kist.re.kr (G.H.); jl5387@kist.re.kr (J.W.L.); kim@kist.re.kr (K.K.); ikwon@kist.re.kr (I.C.K.); 2 Department of Pharmaceutical Engineering, Dankook University, Cheonan 31116, Korea; 3 KU-KIST Graduate School of Converging Science and Technology, Korea University, Seoul 02841, Korea; 4 Department of Cancer Biology, Dana-Farber Cancer Institute, 450 Brookline Avenue, Boston, MA 02215, USA

**Keywords:** extracellular vesicle, inflammatory disease, EV engineering, biomarker

## Abstract

Extracellular vesicles (EV) deliver cargoes such as nucleic acids, proteins, and lipids between cells and serve as an intercellular communicator. As it is revealed that most of the functions associated to EVs are closely related to the immune response, the important role of EVs in inflammatory diseases is emerging. EVs can be functionalized through EV surface engineering and endow targeting moiety that allows for the target specificity for therapeutic applications in inflammatory diseases. Moreover, engineered EVs are considered as promising nanoparticles to develop personalized therapeutic carriers. In this review, we highlight the role of EVs in various inflammatory diseases, the application of EV as anti-inflammatory therapeutics, and the current state of the art in EV engineering techniques.

## 1. Introduction

Extracellular vesicles (EVs), including exosomes and microvesicles, are secreted by almost all types of cells and carry genetic information between cells. Thus, EVs emerge as important contributor in cell–cell signaling and communication between cells. These EVs are characterized according to their size, with exosomes ranging from 50 to 100 nm and microveisicles usually larger than 200 nm [1,2]. EVs are able to transfer variety of cargos including proteins, lipids, antigen and RNAs such as miRNA, siRNA [2,3,4]. In addition, it has been reported that chromosomal DNA is present in certain types of EVs [5,6]. EVs have been reported to have a variety of functions and, in particular, contribute to antigen-specific and non-specific immune regulation to both immune and non-immune cells in the form of immunomodulation [7,8]. They also likely play an important role in modulating inflammatory diseases, such as cardiovascular disease, dermatitis, diabetes, and arthritis [9,10].

With their given ability to modulate inflammation, EVs have tremendous potential as therapeutic agents and biomarkers. Since EVs have several advantages such as biocompatibility, low toxicity, low immunogenicity, and membrane permeability, they are considered as an ideal engineerable nanotherapeutic carrier for delivering various drugs and targeting inflammation. In this review, we focus on the role of EVs in various inflammatory diseases and their potential as therapeutic agents, and outline at the latest advances in EV engineering technologies for targeting and modulating inflammation.

## 2. The Role and Therapeutics Potential of Extracellular Vesicles in Inflammatory Diseases

Recent research on the utility of EVs in inflammatory diseases is being studied in two broad fields. Currently, many researchers believe that EVs could be used as a potential biomarker for a liquid biopsy, an alternative to a typical biopsy characterized by various limitations, through studies of EV’s role in inflammatory diseases [11,12,13]. On the other hand, it is suggested that EVs can also be utilized as therapeutic vehicles and targets for the treatment and prevention of inflammatory diseases. This section will focus on the role of EVs involved in the immune response, either directly or indirectly in inflammatory diseases, and discuss the therapeutic utility of various EVs (Table 1).

### 2.1. Cardiovascular Diseases

#### 2.1.1. Role of Extracellular Vesicles in Cardiovascular Diseases 

Cardiovascular diseases (CVDs) are caused primarily by atherosclerosis, a chronic inflammatory disease in which the vascular endothelium is constantly damaged, causing endothelial dysfunction [41,42]. EVs of various cells such as leukocytes, platelets, smooth muscle cells and endothelial cells are involved in all stages of vascular inflammation, including endothelial activation, monocyte adhesion and transmigration, resulting in atherosclerosis [9,43,44]. Indeed, it has been reported that EVs can induce the release of pro-inflammatory cytokines, such as interleukin (IL)-6 and IL-8, from endothelial cells or leukocytes [45,46]. In addition, there have been reports that platelet-derived EVs enhance leukocyte adhesion by delivering pro-inflammatory molecules such as caspase-3 and RANTES protein to endothelial cells [47,48]. In view of these findings, it has been shown that circulating EV levels are associated with the occurrence of major cardiac dysfunction. Research on EVs as a prognostic and diagnostic biomarker is being actively conducted by improved EV purification technology and accurate content quantitative analysis technology.

#### 2.1.2. Therapeutic Potential of Extracellular Vesicles for Cardiovascular Diseases

Inflammatory responses in CVDs have become a target for cardioprotection. After myocardial infarction (MI), immune cells infiltrate the infarcted area to remove the wounded tissue and participate in cardiac repair [43,44]. EVs affect cardiac repair by interacting with these invasive immune cells and modulating the polarization of immune cells and their secretion of cytokines. For example, cardiosphere-derived cell (CDC)-derived EVs loaded with Y-RNA fragments improved cardiac repair by increasing the release of IL-10 in the infarcted myocardium [14]. In addition, dendritic cell-derived EVs reduced the expression of pro-inflammatory cytokines by activating CD4-positive T cells [15]. Furthermore, several studies have been conducted to utilize EVs as drug delivery vehicles by overexpressing miRs, such as miR-93 and miR-181, which are related to cardiac repair, through genetic modification of the parental cells of EVs [16,17]. There have also been attempts to encapsulate EVs with functional peptide hydrogels in order to prolong EVs retention in the heart more than free EVs [18]. Despite the promising perspective on CVD treatment, EV-based therapies still need further investigation from experimental data to clinical application. Through a clear understanding of the mechanisms of EVs and controlling the balance between the harmful and beneficial effects of EVs in the heart, EVs could be utilized as new therapeutic tools to combat CVDs.

### 2.2. Skin Inflammation

#### 2.2.1. Role of Extracellular Vesicles in Inflammatory Skin Disorders

Psoriasis, atopic dermatitis (AD), systemic lupus erythematosus (SLE), and chronic wound healing are refractory chronic inflammatory skin diseases. Inflammatory skin disorders that proceed through complex pathophysiological processes are also affected by EVs derived from various cells. First, psoriasis is the most common chronic inflammatory skin disease caused by abnormal differentiation and growth of keratinocytes and a large number of infiltrated immune cells [49]. Many studies have shown that endothelial and platelet-derived EVs are elevated in patients with psoriasis [50,51,52]. Recently, potential biomarkers of psoriasis are being discovered through miRNA profiling analysis of plasma-derived EVs [53,54]. AD, one of the eczemas, is another common chronic inflammatory skin disease. Due to the findings that AD patients, characterized by dysfunction of the skin barrier, are susceptible to Staphylococcus aureus, several studies have explored the correlation between Staphylococcus aureus-derived EVs and AD. For example, it has been reported that Staphylococcus aureus-derived EVs increase the production of pro-inflammatory cytokines in dermal fibroblasts, activate endothelial cells, and induce monocyte recruitment [55,56]. In addition, EVs derived from thymol-treated *Staphylococcus aureus* showed their therapeutic potential for AD by alleviating skin lesions in AD [57]. SLE is a chronic inflammatory disease caused by the production of various autoantibodies due to viral infection, hormonal abnormalities etc. It harms multiple organs, and skin is the second most commonly affected organ [58]. According to an earlier reported study, the levels of EVs derived from annexin V non-binding cells were high in SLE patients, and the levels of IgG, IgM, and C1q in EVs were elevated [59,60]. A recent study reported that distinct subpopulations of EVs may have different functions, and that there is an increase in mitochondrial-carrying IgG-positive large EVs in SLE patients [61]. Wound healing usually occurs in four stages: hemostasis, inflammation, proliferation and maturation. Chronic wound healing, which is common among diabetics and older people, is mostly stuck in the inflammatory phase and delayed wound healing. Numerous studies on EVs derived from various types of cells involved in the wound healing process have been reported [62,63,64]. Studies have shown that the expression level of miR-21 in EVs derived from keratinocytes in a mouse diabetes model is remarkably low [65]. In addition, there have been studies showing that advanced glycation end products induce human umbilical endothelial cells to secrete miR-106b-rich EVs, thereby reducing collagen synthesis and delaying skin wound healing [66].

#### 2.2.2. Therapeutic Potential of Extracellular Vesicles for Inflammatory Skin Disorders

In dermatology research, EVs are starting to show promising prospects as a therapeutic for inflammatory skin diseases. Several studies have shown that EVs can be used as a therapeutic agent for wound healing. Umbilical cord blood-derived EVs rich in miR-21 accelerated cutaneous wound healing by promoting angiogenesis of endothelial cells and enhancing the migration and proliferation of fibroblasts [67]. Keratinocyte-derived EVs also facilitated wound healing by promoting angiogenesis and regulating fibroblast function through miR-21 [20]. Recently, a new approach to chronic inflammation treatment by combining these therapeutic EVs and biomaterials is being studied. A study has been reported to promote skin regeneration and accelerate wound healing through injectable antibacterial hydrogels with EVs derived from mesenchymal stem cells (MSCs) [21]. In addition, a study also reported that mononuclear cell-derived EVs carrying various miRs with a light-activatable hydrogel could promote wound healing [22]. EVs, especially exosomes, can also be used as drug delivery vehicles for inflammatory skin diseases. Choi et al. incorporated srIκB (super-repressor IκB) protein into exosomes by optogenetic method to inhibit the action of nuclear factor kappa-light-chain-enhancer of activated B cells (NF-κB), which plays a major role in activating inflammation [68]. However, these drug loading and targeting techniques must be applied in appropriate situations to suit their respective advantages, and standardization and deep understanding of techniques are also required to apply them to clinical trial.

### 2.3. Autoimmune Diseases

#### 2.3.1. Role of Extracellular Vesicles in Autoimmune Diseases

Autoimmune disease refers to diseases such as rheumatoid arthritis (RA), multiple sclerosis (MS), and type 1 diabetes (T1D) caused by the self-immune system responding to autoantigen. There are many reports that the level of circulating EV increases in an autoimmune condition [69,70]. RA is a chronic inflammatory disorder that primarily affects joints. The pathogenesis of RA may be related to EVs involved in many complex cell-to-cell communications such as antigen presentation and inflammation [69,71]. It is known that synovial cell-derived EVs activate surrounding cells to secrete pro-inflammatory mediators for damaging cartilage [72]. MS, also known as encephalomyelitis, is an inflammatory demyelinating disease in which the insulating covering of nerve cells in the central nervous system is damaged by autoimmune activated immune cells [73,74]. It has been reported that EVs can transfer brain antigens to periphery through the blood brain barrier (BBB) [75]. T1D, formerly referred to as juvenile diabetes, is a form of diabetes in which little or no insulin is produced because autoimmunity destroys β cells of the islet of Langerhans in the pancreas. Recent studies reported that EVs deliver autoantigen peptides involved in the pathogenesis of T1D to insulin-producing β cells. A recent study reported that EVs deliver autoantigen peptides involved in the pathogenesis of T1D to insulin-producing β cells, inducing apoptosis and resulting in insulin secretion disorders.

#### 2.3.2. Therapeutic Potential of Extracellular Vesicles for Autoimmune Diseases

Receptors for pro-inflammatory cytokines such as TNF-α have also been reported to be found on the EV surface [76]. These findings suggest that EVs may act as an endogenous mechanism for receptor transfer to poorly expressed recipient cells or for the inhibition of inflammation by the release of decoy receptors. Thus, despite the major pathogenic role of EVs in autoimmune diseases, many researchers are currently attempting to utilize these bioparticles for therapeutic agents. Kim et al. reported that IL-10-treated dendritic cell-derived EVs can suppress the onset of arthritis and reduce the severity of established arthritis [23]. In addition, MSC-derived microparticles and exosomes both play an immunosuppressive role in inflammatory arthritis, but exosomes were more effective [24]. Based on the role of EVs in MS, several groups have developed therapeutic strategies that use the function of EVs. MSC-derived EVs stimulated with IFN-γ carried anti-inflammatory factors and neuroprotective proteins and showed a good therapeutic effect on MS [25]. EVs derived from dendritic cells overexpressing TGF-β1 have been shown to reduce the expression of MS by inhibiting Th1 and Th17 differentiation and promoting Treg production [26]. In addition, there has been a study to engineer a mouse microglial cell line to secrete exosomes that carry the anti-inflammatory cytokine IL-4 and overexpress the “eat me” signal MFG-E8 on its surface [27]. Zhuang also reduced neuroinflammation by loading curcumin or a signal transducer and activator of transcription 3 (Stat3) inhibitor into EVs and delivering them to microglia cells through a non-invasive intranasal route [39]. In treating T1D, EVs also have shown therapeutic potential. A study reported that EVs isolated from urine-derived stem cells can prevent kidney injury from diabetes by inhibiting apoptosis of podocytes and promoting angiogenesis [28]. Also, Lee et al. showed that MSC-derived EVs inhibit the activity of antigen-presenting cells and suppress the development of T helper cells, thereby reducing the immune response in T1D and uveitis animal models [29,30]. Collectively, with an understanding of the underlying mechanisms and roles of EVs associated with each stage of the disease, EVs can be used as a potential therapeutic tool for autoimmune diseases.

### 2.4. Respiratory System Inflammation

#### 2.4.1. Role of Extracellular Vesicles in Respiratory System Inflammation

Pulmonary inflammatory diseases can be categorized as either acute or chronic. Acute inflammation entails vasodilation and recruitment of immune-modulating cells at the site of the injury, while chronic inflammation is commonly characterized by fibrosis, a scarring of the lung tissue from a persistent inflammation [77,78]. In an acute inflammatory setting, like that of acute lung injury (ALI) and its more severe form (ARDS), inflammation causes a profound immune response throughout the body and the increased level of pro-inflammatory cytokines [79]. The role of EVs in reinforcing the process of inflammation has been extensively studied both in vitro and in vivo. Secreted EVs enable intercellular communication between damaged cells of lung tissue and alveolar macrophages or between alveolar macrophages [80,81]. Analysis of EVs from a bronchoalveolar fluid (BALF) expressed a high level of inflammatory molecules, both major histocompatibility complex (MHC) [82] and various miRNAs [83,84]. The contents and the main cellular source of BALF-EVs, however, varied based on the conditions and the type of stimulus. Depending on the mode of induction, the sterile method (toxicant, acid, or hypoxia) induced BALF-EVs and those induced by the non-sterile method (LPS or bacterial infection) vary in the major secreting cells as well as the interleukin profile within the BALF-EVs [85]. Each stimulus can induce micro-vesicles with different contents, and therefore, activates different pathways in receiving cells [81]. BALF-EVs from controlled asthmatic patients showed different miRNAs profiles control healthy subjects [83,86]. Exhaled breath condensates EVs from COPD, asthma, healthy individuals showed different miRNA expression: some were down-regulated specifically to asthmatic patients (miR-1248, miR1291, and let-7a), while some were down-regulated in both COPD and asthmatic group (miR-328 and miR-21) [87,88]. In such a way, miRNAs in the EVs can serve as diagnostic and prognostic biomarkers for lung inflammation. For instance, let-7c and miR-125b from EVs are found to be inversely related to ARDS severity [31]. Besides miRNA analysis, levels of circulating EV can serve as the prognostic biomarkers of pulmonary inflammatory disease [89]. Increased circulating EVs were affiliated with a lower risk of ARDS in critically ill patients, thus providing insights into health and disease progression. The cellular origin of the EVs can also serve as the biomarker for ALI/ARDS: ARDS is associated with increased leukocyte-derived EVs, while ALI is associated with increased alveolar EVs [90].

#### 2.4.2. Therapeutic Potential of Extracellular Vesicles in Respiratory System Inflammation

Mesenchymal stem cell therapy is regarded as a promising treatment option for both chronic and acute lung diseases [91]. Its potential application in tissue repair and suppression of inflammation has gained great attention. Some pulmonary inflammatory diseases, like ARDS/ALI, still have no treatment options [33]. MSC therapy showed promising results for ARDS/ALI, which raised high excitement and led to the development of several MSCs treatment agents [92]. In recent findings, immunomodulating effects of MSCs were attributed to MSC-secreted EVs [32]. Having comparative immune suppressive effects to MSC [34], MSC-EVs have several advantages over MSC as a therapeutic agent. MSC-EVs have a higher safety profile and lower immunogenicity; MSC-EVs could also avoid several unwanted side effects of MSC, such as immune rejection, clogging pulmonary capillaries, and stem cell-induced tumor formation [90,93]. MSC-EVs modulate inflammation in multiple pathways [35]. MSC-EVs protect epithelial cells from reactive oxidative species (ROS) by delivering miRNA 21-5p, which inhibits ROS-induced apoptotic pathways [31]. Besides anti-apoptotic miRNA, MSC-EVs contain anti-microbial proteins against Gram-negative bacteria. MSC-EVs increase the phagocytotic activity of neutrophils and monocytes; hence, bacterial pneumonia was less severe for the MSC-EVs treated group [32]. During the inflammatory phase, MSC-EVs induce an increase in phagocytosis; however, in a setting of resolution, MSC-EVs induce the alveolar macrophage polarization to M2 macrophages, which are commonly associated with wound healing and anti-inflammatory cytokines [33]. Thus, levels of immunosuppressive cytokines, like IL-10 and TGF-beta, increased while pro-inflammatory cytokines, like IL-8, IL-1 beta, and TNF-alpha, decreased [34]. Due to the high concentration of anti-inflammatory cytokines, dendritic cell maturation is suppressed, and it leads to additive anti-inflammatory effects [35]. For adaptive immunity, MSC-EVs inhibit B cell proliferation and differentiation, and they promote the differentiation of T helper cells to regulatory T cells, which causes an increase of anti-inflammatory cytokines (TGF-beta, IL-10, and PEG_2_) and a decrease of pro-inflammatory cytokines (IFN-gamma, TNF-a, and IL-1 beta) [34].

### 2.5. Neuroinflammation

#### 2.5.1. Role of Extracellular Vesicles in Neuroinflammation 

EVs have both beneficial and detrimental roles in neuroinflammation. EVs can facilitate neuroinflammation by spreading the inflammatory signals from damaged cells to neighboring naïve neural cells [94]. The resident phagocytic cells within the brain, microglial cells, are known to be the first to respond in terms of neuroinflammation [95]. Once activated, microglial cells multiply in numbers, and each releases a greater number of ATP-filled EVs, sending danger signals across the brain. Thus, nearby microglial cells and astrocytes are stimulated and produce secrete inflammatory signals which further disseminate the inflammation. Besides worsening the inflammation via proinflammatory contents, EVs play crucial roles in several neurodegenerative disease progression, and consequently, lead to more pronounced chronic neuroinflammation [96]. Neural diseases, such as Alzheimer’s disease, Parkinson’s disease, traumatic brain injury (TBI), and multiple sclerosis (MS), all involve neuroinflammation and loss of cognitive function as the results [97]. The role of EVs as carriers of pathogenic proteins applies to Alzheimer’s disease (AD), Parkinson’s disease (PD), Creutzfeldt–Jacob disease (CJD), and amyotrophic lateral sclerosis (ALS) [98,99,100]. In the case of AD and PD, the main destructive effects are induced by the spreading of the causative agents: αβ peptide and phosphorylated tau for AD, α-Syn for PD; in the case of AD, ab protein aggregates formed extracellularly occurs as the cells get rid of ab with EVs. Meanwhile, through EVs, prion protein (PrP^c^) is carried across the brain, leading to overall damage [101]. Besides causing the conformational change of protein, these EVs contained higher levels of mRNAs that are related to neural disorders (miRs-29b, 128a, 146a) [97]. The specific interplay between neurodegenerative disease and neuroinflammation needs to be further clarified, but the interdependent relationship between neuroinflammation and the progression of neurodegenerative disease is confirmed in many recent studies [102,103,104]. As EVs move across the blood–brain barrier (BBB), transport of inflammatory materials from the peripheral to the brain can induce neuroinflammation, and in reverse, inflammation from the periphery can be the cause of neuroinflammation as well. HIV and HCV, typified by chronic peripheral inflammation, are associated with a higher rate of neurological problems. Individuals who suffered systemic autoimmune disease, also, showed a higher rate of the neurological disorder compared to healthy individuals [105].

#### 2.5.2. Therapeutic Potential of Extracellular Vesicles in Neuroinflammation

EVs derived from MSC demonstrated its neuroprotective effects in several preclinical models [106]. In the preterm brain injury model, MSC-EV therapy reduced the rate of a histological sign of neuroinflammation and improved the inflammation-induced neuronal degeneration [107]. In the ischemic stroke model of rats, intra-arterial injection of MSC-EV has been shown to decrease the cell activation of astrocytes, microglia, and infiltrating leukocytes [36]. MSC-EV therapy leads to suppression of pro-inflammatory cytokines, TNF-α and IL-1b, and reduced secretion of anti-inflammatory cytokines, IL-10 and TGF-β [35]. Besides their neuroprotective effects, EVs have shown potential as drug delivery vehicles due to their safety and targetability based on their origin [108]. Especially in neural disease, crossing BBB remains a challenge for potential promising therapies. As intravenous EV injection has been found to cross BBB and target brain cells [37], MSC-EV can serve as the optimal carrier for various neuroinflammatory diseases. Curcumin-loaded EVs have demonstrated neuroprotective effects in the ischemia-reperfusion injury model and LPS induced brain inflammation [38]. In the setting of LPS induced brain inflammation model, EVs packaged with curcumin have been delivered intranasally and efficaciously induced selective curcumin-loaded EV uptake by activated resident immune cells and induced apoptosis in those pro-inflammatory neural cells [105]. Besides loading anti-inflammatory chemical compounds, MSC-EVs have been utilized extensively as the delivery vehicle for gene therapy to the brain [37]. Specifically, MSC-EV mediated transport of miR-124 is found to reduce the neuroinflammation: in cocaine mediated brain inflammation, intranasal delivery of miR-124 loaded EVs to the brain dampened the secretion of proinflammatory cytokines (TLR4, STAT3, NF-kB p65, and MYD88) and ceased the activation of microglia [40]. A clinical trial of MSC-EVs for brain inflammation is currently ongoing for acute ischemic stroke (NCT03384433). However, further pre-clinical and clinical studies need to be done to determine the full potential of MSC-EV usage in brain inflammation [106].

## 3. Surface Engineering Techniques of EVs

**Scheme 1:**
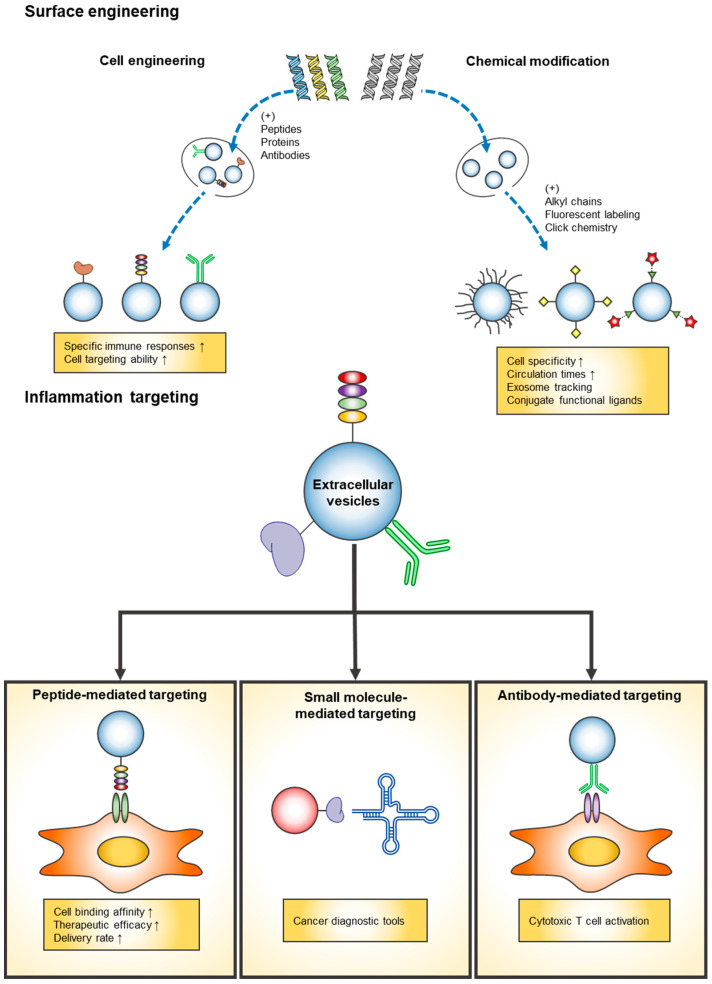
Surface engineering techniques of EVs and targeting strategies for inflammation. Cell engineering methods that express peptides, proteins and antibodies on the EV surface could enhance specific immune responses and cell targeting capabilities. The method of attaching various substances through chemical modification could provide new functions different from normal EV. These EVs could achieve increased cell specificity and circulation times. In addition, it can be applied to inflammation by activating immune cells with peptides or antibodies expressed on the EVs surface, and by diagnosing cancer proteins on the EVs surface using aptamers. Attachment of specific peptides to EVs increases cell binding affinity, therapeutic efficacy, and delivery rate, and attachment of specific antibodies can lead to activation of cytotoxic T cells.

The surface modification of EVs is a necessary step for practical applications because surface engineering brings additional functionality to EVs and allow targeting, enhanced intracellular uptake, and prolonged circulation time. There are two main approaches that classify surface engineering (Scheme 1).

### 3.1. Cell Engineering

Cell engineering is a method that introduce peptides, proteins, and antibodies on the surface of EVs. The cell surface is engineered by fusing proteins of interest, introducing targeting peptides, or immobilizing proteins to the inner surface of EVs [109,110].

Fusion of proteins with the Lactadherin C1C2 domain has been found to bind exosome lipids [111]. In more detail, the C1C2 domain of Lactadherin provides exosome targeting strategy, where fusion proteins to the C1C2 domain of Lactadherin generates chimeric proteins that bind to exosome lipids. Thus, exosome enables the production of multiple copies of antigens bounding particles and provides immune system that can react to encounter foreign microorganisms. Hatman et al. also coupled carcinoembryonic antigen (CEA) and HER2 to the C1C2 domain of Lactadherin and found that the fusion proteins showed enhanced expression in exosomes and antigen specific immune responses for enhanced therapeutic anti-tumor effects [112].

Genetic engineering is able to introduce targeting peptides on the surface of EVs. The Lamp2b is a protein that found in exosomal membranes and one of the proteins that has been and widely applied with targeting peptides [37,113]. For example, targeting was achieved by fusing Lamp2b to the neuron specific rabies viral glycoprotein (RVG) peptide [37]. When the Lamp2b constructs were transfected to dendritic cells, Lamp2b was strongly expressed in dendritic cells and the constructs successfully endowed exosomes with cell targeting ability. In addition, Lamp2b was fused to αv integrin-specific iRGD peptide to facilitate tumor targeting ability by engineering immature mouse dendritic cell line (imDC) [114]. The engineering of imDCs expressed Lamp2b and the iRGD exosomes from imDCs loaded with DOX showed highly efficient targeting and demonstrated enhanced DOX delivery to αv integrin-positive breast cancer cells.

Genetic engineering can be also applied to immobilize proteins to the inner surface of exosomes. For example, cryptochrome 2 (CRY2)-conjugated cargo proteins to the exosomes were designed by encoding CIBN to an exosome-associated tetraspanin protein CD9 with blue light illumination [115]. When the cargo proteins are introduced into the exosomes via biogenesis, it is detached from CD9-conjugated CIBN by light illumination and release into the exosomal intraluminal space and efficiently deliver to target cells. Thus, protein-loaded exosomes demonstrated enhanced intracellular levels of cargo proteins and their function in recipient cells. Shalitin et al. also reported that CRY2 undergoes a blue-light-dependent phosphorylation and proposed that the absorption of photons by a cryptochrome undergoes its conformation change and triggers signal transduction and physiological responses [116].

### 3.2. Chemical Modification

One way to anchor molecules into the lipid bilayer of exosomes are using alkyl chains through hydrophobic interactions. Koojimans et al. decorated EVs with targeting ligands conjugated to polyethylene glycol (PEG). The introduction of PEG-conjugated nanobodies to EVs provided cell specificity and prolonged circulation times while unmodified EVs rapidly cleared from the circulation in in vivo studies [117]. In addition, exosomes were labelled using the PKH26 Red Fluorescent [118]. When exosomes derived from mammary epithelial cells labelled with PKH26 were incubated with tumor cells, the exosomes were efficiently incorporated into the tumor cells. Exosomes isolated from bladder cancer were labelled with PKH26 and showed that exosome uptake based on the number of PKH26 positive spots is dose and time dependent [119]. Smyth et al. also demonstrated DIR incorporated into exosomes, where DIR is fluorescent that label exosomes [120]. They labelled exosomes with the lipophilic fluorescent tracer DIR and used them to study exosomes’ rates of clearance and biodistribution. These studies were performed to stain exosomal membranes by the intercalation of aliphatic chains into lipid bilayers.

In addition, there are another method for conjugation of ligands to the exosome surface via click chemistry. Click chemistry, also called Copper-catalyzed azide alkyne cycloaddition, is a highly efficient reaction that forms a thiazole linkage between an alkyne and an azide group [121]. Thus, the alkynes are first grafted to the EV membrane and then react with azide group of molecules of interest and form azide-alkyne cycloaddition. Presolski et al. also used the click chemistry for the coupling of cargo-azide to biomolecule-alkyne and designed azaide-modified fluorophores [122]. Click chemistry was also used to conjugate functional ligands onto exosomal surfaces and Tian’s group proposed the cyclo(Arg-Gly-Asp-D-Tyr-Lys) peptide that shows high affinity to integrin αvβ3 was conjugated on mesenchymal stromal cell (MSC)-derived exosomes [123].

## 4. Strategies for Inflammation Targeting EVs

EVs have been decorated with various targeting ligands including peptides, small molecules, antibodies, and aptamers to obtain active targeting. EVs are modified to target inflammatory cells for anti-inflammation therapy.

### 4.1. Peptide-Mediated Targeting

The cyclo(Arg-Gly-Asp-D-Tyr-Lys) peptide has been conjugated on the surface of exosome via bio-orthogonal chemistry to target the lesion region of the ischemic brain [123]. The MSC-derived exosomes conjugated with peptide showed high affinity to integrin αvβ3 in reactive cerebral vascular endothelial cells. Wang et al. designed exosomal enriched membrane protein (Lamp2b) fused to ischemic myocardium-targeting peptide CSTSMLKAC (IMTP) and found MSC-derived IMTP-exosome exhibited enhanced therapeutic efficacy on acute myocardial infarction [67]. In addition, exosome expressing cardiac-targeting peptide (CTP)-Lamp2b were generated by Kim’s group and CTP-exosome showed 15% enhanced delivery of exosomes to the heart cell and heart tissue of mice [124]. Zhang et al. used cyclo(1,12)PenITDGEATDSGC (cLABL) peptide that is known to bind and upregulate intercellular cell-adhesion molecule-1 (ICAM-1) on HUVEC [125]. They demonstrated the inflammation-targeting peptide is inducing inhibition of the infiltration of immune cells via blockage or internalization of ICAM-1 receptors on HUVEC. Vascular cell adhesion molecule-1 (VCAM-1) peptide was also proposed as an inflammation-targeting peptide to identify the inflammatory activation of cells involved in atherosclerosis [126].

### 4.2. Small Molecule-Mediated Targeting

Small molecule targeting ligands are referred to targeted delivery that have an average molecular weight less than 1 kDa. The small molecule-based targeting strategy has been applied in EV-based therapies. Pi et al. developed siRNA-loaded nanoparticles and displayed folate on the outer surface of the EVs [127]. Folate is known as an attractive targeting ligand since many cancers overexpress folate receptors [128]. They demonstrated that folate displayed EVs inhibited colorectal cancer growth in mice. In addition, monosaccharides such as glucose and galactose are another class of small molecule targeting ligands. Glucose targets the GLUT1 receptor that is overexpressed at the blood–brain barrier and its derivative modified therapeutic modalities have been studied for glioma targeting [129].

As a low molecular weight targeting system, aptamer was also used in several studies for aptamer-mediated inflammation targeting strategy. Aptamers also known as chemical antibodies can function as therapeutic and diagnostic tools due to its target-binding, no immunogenicity, and deep tumor penetration [130]. Aptamer-guided exosome capturing nanoplatform system has been developed as a diagnostic tool. For example, aptamer was utilized to capture HSP70 positive exosomes in urine samples taken from breast, ovarian, or lung cancer patients [131]. Li et al. designed an exosome that targets dendritic cells (DC-Exosome). MSCs derived exosomes was applied to the mTOR siRNA delivery system of targeting dendritic cells. Aptamer/siRNA chimera performed the targeted delivery of microRNAs into DCs and maintained long-term immune tolerance [132].

### 4.3. Antibody-Mediated Targeting

Antibodies for immune cells or tissue cells are also introduced on the surface of EVs, which allows specific inflammation targeting. Exosomes can be act as artificial cellular immunity controller to activate cytotoxic T cells for cancer cell killing by modification via antibody fusion [133]. Exosome fused with antibody was called synthetic multivalent antibodies retargeted exosomes (SMART-Exos) and provided a versatile platform technology for immunotherapy as well as a potent anticancer immune response.

## 5. Challenges and Perspectives

EVs have great interest and importance in therapeutic applications for the clinic due to biocompatibility as a nature nanoplatform. They have exhibited therapeutic potential in various inflammatory diseases including autoimmune diseases, neurology diseases, and myocardial diseases. To date, several phase I or phase II clinical trials have been conducted based on considerable evidence of their therapeutic utility accumulated in preclinical studies (Table 2). However, the practical therapeutic application of EVs faces significant challenges. The major challenges that must be addressed before turning EVs to the clinic are: (i) large-scale production, (ii) content analysis of EVs and (iii) EVs engineering technology control. The separation of EVs from cell culture media is carried out by methods such as laboratory scale ultracentrifugation or filtration. The yield of EV obtained from these methods may vary slightly depending on the type of cultured cells, but is generally very poor. Therefore, standard EVs production methods need to be developed for large scale production, rapid isolation, and quality control. Also, the analysis of EVs is focused on the content of the EVs. Cell-derived EVs represent the properties such as nature and function of the donor cells. However, until now, there has been only a little way to know how to represent the properties. Prior to the engineering of EVs, the content should be characterized in the contexts of EVs heterogeneity. In therapeutic applications of EVs, it may be necessary to discover and recognize the contents that cause therapeutic effects or side effects. Also, content analysis needs to be linked to standards for the evaluation of EVs. Through the overall content analysis and evaluation standard, the specific donor cells can be selected for the treatment of the specific diseases. In terms of engineering technology, therapeutic molecules, including chemical drugs, genetic material, and proteins, have been loaded on EVs. During the engineering process, the loading efficiency of cargo to be loaded onto EVs should be controllable and should not affect the inherent characteristics of EVs. Changes in the size and surface potential of EVs can affect the biological activity and therapeutic efficacy of EVs. 

The field of EVs therapeutics has been rapidly growing with advanced nanotechnology. As a natural nanoplatform, EVs provide a powerful option for overcoming the challenges of inflammatory diseases, including potential toxicity and complexity. EVs engineered with nanotechnology have shown enhanced therapeutic efficacy. Especially, recent studies for selective drug encapsulation were focused on the sorting machinery working in EVs biogenesis and release. Therefore, uncovering the biological pathway related to the packaging of EVs may provide us with the novel tools for improving EVs-based therapy. In addition, a light-triggered drug loading technique was developed by integrating optically light reversible protein interaction module and EVs biogenesis and release process. [115]. This research may suggest the importance of conversing technology with other research fields such as mechanics, electronics, optics, and omics. The future application of EVs in the detection and treatment of various inflammatory diseases will be possible through deep and broad collaboration between different fields.

## Figures and Tables

**Table 1:** Summary of therapeutic potential of extracellular vesicles according to disease.

Disease	EV Source	Effective Molecule	Therapeutic Effect	Target Cell	References
**Cardiovascular diseases**					
Cardiovascular diseases	Cardiosphere-derived cell	Y RNA fragment	Increase the expression of IL-10	Cardiomyocyte	[14]
Cardiovascular diseases	Dendritic cell	Not determined	Activate CD4+T cell	CD4+T cell	[15]
Cardiovascular diseases	Adipose-derived stromal cells & mesenchymal stem cell	miR-93-5p & miR-181a	Suppress inflammation	Hypoxic H9c2 cell & Peripheral blood mononuclear cell	[16,17]
Cardiovascular diseases	Human umbilical cord mesenchymal stem cell	PA-GHRPS peptide	Protect from oxidative stress	H9C2 cell	[18]
**Skin inflammation**					
Cutaneous wound	Human umbilical cord blood	miR-21-3p	Promote wound healing factors	Human skin fibroblast	[19]
Skin wound	Human keratinocyte	miR-21	Mediate a pro-inflammatory response	Human foreskin fibroblast	[20]
Chronic diabetic wound	Adipose-derived mesenchymal stem cell	Not determined	Promoted proliferation and angiogenesis	Human umbilical vein endothelial cell	[21]
Chronic diabetic wound	Human umbilical cord blood mononuclear cell	miR-150-5p	Increase skin neovascularization and re-epithelization	Human umbilical vein endothelial cell & normal dermal human fibroblast	[22]
**Autoimmune diseases**					
RA	IL-10-treated dendritic cell	Not determined	Suppress inflammation	Not determined	[23]
RA	Mesenchymal stem cell	Not determined	Inhibit T lymphocyte proliferation & decrease the percentage of CD4+ and CD8+ T cell subsets	T lymphocyte & CD4+ and CD8+ T cell	[24]
Autoimmune & Neurodegenerative diseases	IFNγ-treated mesenchymal stem cell	IFN-γ & anti-inflammatory RNAs	Decrease pro-inflammatory cytokines & increase immunosuppressive cytokines	CD4+CD25+FOXP3+ regulatory T cells & peripheral blood mononuclear cell	[25]
EAE	Gene-modified dendritic cell	TGF-β1	Decrease the frequency of Th17 cell & inhibit proteolipid protein	Th17 cell & CD4+T cell	[26]
MS	Engineered a murine microglia cell	IL-4	Increase anti-inflammatory factors	Macrophages & microglia	[27].
T1D	Human urine-derived stem cell	Not determined	Protect target cell from apoptosis	Human podocyte cell & tubular epithelial cell	[28].
T1D	Mesenchymal stem cell	Not determined	Inhibit activation of antigen-presenting cell & suppress development of Th1 and Th17 cell	Not determined	[29,30].
**Respiratory system inflammation**					
Lung injury	Mesenchymal stem cell	miR-21-5p	Inhibits ROS-induced apoptotic pathway	Epithelial cell	[31]
Bacterial pneumonia	Mesenchymal stem cell	Not determined	Increase the phagocytotic activity	Neutrophil & monocyte	[32]
Lung injury	Mesenchymal stem cell	Not determined	Polarize into M2 macrophages	Alveolar macrophage	[33]
Lung injury	Mesenchymal stem cell	Not determined	Reducing the pro-inflammatory cytokines	Regulatory T cell	[34]
Lung fibrosis	Mesenchymal stem cell	Not determined	Suppress the maturation	Dendritic cell	[35]
**Neuroinflammation**					
Ischemic stroke	Mesenchymal stem cell	Not determined	Decrease the cell infiltration	Astrocyte & leukocyte	[36]
Alzheimer’s disease	Bone marrow stem cell	BACE1 siRNA	Reduce the expression of disease-related proteins	Neuro2A cell	[37]
Brain inflammation	Embryonic stem cell	Curcumin	Reduce inflammation and N-methyl-d-aspartate receptor expression	Not determined	[38]
Brain inflammation	EL-4 cell	Curcumin	Decrease the percentage of CD45.2+IL-1β+ cell	Not determined	[39]
Neuroinflammation	Dendritic cell	miR-124	Alleviate cocaine-mediated microglial activation	Microglia	[40]

**Table 2:** Human clinical trials of EVs in inflammatory diseases.

Disease	Phase	Source of Exosomes	Results and Status	NCT Numbers
Cutaneous wound healing	Phase I (*n* = 5)	Plasma derived exosomes	Enrolling	NCT02565264
Skin graft	Phase I (*n* = 24)	Platelet derived extracellular vesicles	Enrolling	NCT04664738
Diabetes Mellitus Type 1	Phase II, III (*n* = 20)	Umbilical cord blood derived mesenchymal stem cell exosomes	Unknown	NCT02138331
Severe Coronavirus pneumonia, ARDS	Phase II (*n* = 60)	Bone marrow derived extracellular vesicles	Not yet recruiting	NCT04493242
Severe Coronavirus pneumonia	Phase I (*n* = 24)	Allogenic adipose mesenchymal stem cell derived exosome	Completed	NCT04276987
Severe Coronavirus pneumonia	Phase II (*n* = 90)	Mesenchymal stem cell derived exosome	Enrolling	NCT04602442
Acute respiratory distress syndrome	Phase I, II (*n* = 169)	Allogenic human mesenchymal stem cell derived exosomes	Not yet recruiting	NCT04602104
Acute Ischemic Stroke	Phase I, II (*n* = 5)	Allogenic mesenchymal stem cell derived exosome	Recruiting	NCT03384433

## Data Availability

Not applicable.

## Abbreviations

EVs	Extracellular Vesicles
PEG	Polyethylene Glycol
MSCs	Mesenchymal Stem Cells
CVDs	Cardiovascular Diseases
IL	Interleukin
AD	Atopic Dermatitis
SLE	Systemic Lupus Erythematosus
RA	Rheumatoid Arthritis
MS	Multiple Sclerosis
T1D	Type 1 Diabetes
ALI	Acute Lung Injury
ARDS	Acute Respiratory Distress Syndrome
BALF	Bronchoalveolar Fluid
COPD	Chronic Obstructive Pulmonary Disease
ROS	Reactive Oxidative Species
AD	Alzheimer’s Disease
PD	Parkinson’s Disease
